# Impact of the COVID-19 Pandemic on Pediatric Surgical Volume in Four Low- and Middle-Income Country Hospitals: Insights from an Interrupted Time Series Analysis

**DOI:** 10.1007/s00268-022-06503-2

**Published:** 2022-03-10

**Authors:** Paul Park, Ruth Laverde, Greg Klazura, Ava Yap, Bruce Bvulani, Bertille Ki, Toussaint W. Tapsoba, Emmanuel A. Ameh, Maryrose Osazuwa, Michele Ugazzi, José Daza, Emma Bryce, David Cunningham, Doruk Ozgediz

**Affiliations:** 1 School of Medicine, University of California, San Francisco, San Francisco, CA USA; 2 Center for Health Equity in Surgery and Anesthesia, University of California, San Francisco, San Francisco, CA USA; 3 Division of Pediatric Surgery, University of Illinois at Chicago, Chicago, IL USA; 4 Department of Surgery, University of California, San Francisco, San Francisco, CA USA; 5 Department of Paediatric Surgery, University Teaching Hospital of Lusaka, Lusaka, Zambia; 6 Department of Paediatric Surgery, Centre Hospitalier Universitaire Pédiatrique Charles De Gaulle, Ouagadougou, Burkina Faso; 7 Division of Paediatric Surgery, National Hospital, Abuja, Abuja, Nigeria; 8 Department of Anesthesia, National Hospital, Abuja, Abuja, Nigeria; 9 Division of Pediatric Surgery, Hospital de los Valles, Quito, Ecuador; 10 Kids Operating Room, Edinburgh, Scotland, UK; 11 School of Medicine, University of California, San Francisco, 513 Parnassus Ave, Suite S-224, San Francisco, CA 94143 USA

## Abstract

**Background:**

The impact of the COVID-19 pandemic on surgical care delivery in low- and middle-income countries (LMIC) has been challenging to assess due to a lack of data. This study examines the impact of COVID-19 on pediatric surgical volumes at four LMIC hospitals.

**Methods:**

Retrospective and prospective pediatric surgical data collected at hospitals in Burkina Faso, Ecuador, Nigeria, and Zambia were reviewed from January 2019 to April 2021. Changes in surgical volume were assessed using interrupted time series analysis.

**Results:**

6078 total operations were assessed. Before the pandemic, overall surgical volume increased by 21 cases/month (95% CI 14 to 28, *p* < 0.001). From March to April 2020, the total surgical volume dropped by 32%, or 110 cases (95% CI − 196 to − 24, *p* = 0.014). Patients during the pandemic were younger (2.7 vs. 3.3 years, *p* < 0.001) and healthier (ASA I 69% vs. 66%, *p* = 0.003). Additionally, they experienced lower rates of post-operative sepsis (0.3% vs 1.5%, *p* < 0.001), surgical site infections (1.3% vs 5.8%, *p* < 0.001), and mortality (1.6% vs 3.1%, *p* < 0.001).

**Conclusions:**

During the COVID-19 pandemic, children’s surgery in LMIC saw a sharp decline in total surgical volume by a third in the month following March 2020, followed by a slow recovery afterward. Patients were healthier with better post-operative outcomes during the pandemic, implying a widening disparity gap in surgical access and exacerbating challenges in addressing the large unmet burden of pediatric surgical disease in LMICs with a need for immediate mitigation strategies.

**Supplementary Information:**

The online version contains supplementary material available at 10.1007/s00268-022-06503-2.

## Introduction

On March 11th, 2020, the COVID-19 outbreak was declared a global pandemic by the World Health Organization [1]. In the following months, there was an unprecedented impact on the provision of surgical care worldwide [2, 3]. As healthcare systems triaged and scrambled for resources during the surges, many surgical activities across different subspecialties were deemed non-essential and put on hold or canceled [4–11]. The additional obstacles to surgical care access have resulted in increased backlogs with substantial economic impact [12–14]. These problems are likely further exacerbated in low- and middle-income countries (LMIC), where the proportion of unmet surgical needs is highest [15, 16].

With children making up about half of the population in LMIC, it has been estimated that at least 6000 children likely died every month during the pandemic due to the collateral effects on health systems [17, 18]. The impact on surgery due to COVID-19 is underreported and poorly characterized. There have been limited published data from LMIC that describe the growing disease burden due to the pandemic [19]. Further data are needed to help address the unmet surgical burden in these regions in order to inform advocacy and mitigation strategies.

## Materials and methods

### Data collection

Kids Operating Room (KidsOR) is a non-governmental organization dedicated to building pediatric operating rooms in LMIC hospitals [20]. As part of the effort, the charity has helped strengthen pediatric surgical data capacity to promote advocacy, quality improvement, and research. Based on a consensus-driven data collection protocol, retrospective and prospective data were collected at partner hospitals (Online Supplementary Table 1). All data were collected and stored on a secure, web-based platform Research Electronic Data Capture (REDCap) [21, 22]. All patients < 18 years old that had operations entered in the database from January 1, 2019, to April 30, 2021, were included. Participants provided informed consent for the collection of data, and the study has been approved by the Institution Review Boards of the University of California, San Francisco (#19-29663).

This multicenter study involved the following partner hospitals: Centre Hospitalier Universitaire Pédiatrique Charles De Gaulle in Ouagadougou, Burkina Faso (“Burkina Faso” hereafter), Hospital de los Valles in Quito, Ecuador (“Ecuador” hereafter), National Hospital in Abuja, Nigeria (“Nigeria” hereafter), and University Teaching Hospital in Lusaka, Zambia (“Zambia” hereafter). Burkina Faso, Nigeria, and Zambia are public tertiary referral hospitals, while Ecuador is private. Out of eight total sites in the database, these four sites were selected based on the criteria of having at least 12 months of data entered in the database before and after March 2020, consistent data collection without gaps during the study period, and signed Memorandum of Understanding (MOU) with KidsOR.

### Data analysis

An interrupted time-series analysis (ITSA) was performed using the two ordinary least-squares regression-based approaches Newey–West standard errors to assess monthly changes in surgical volume, in total and by elective and emergency cases [23]. ITSA was chosen due to its statistical utility in assessing the impact of an event on a population level with a clearly defined time periods that allow for comparing pre- and post-time series data [24]. Pre-COVID-19 period (“pre-COVID” hereafter) was defined as data entered in the 14-month period from January 1, 2019, to February 28, 2020, and post-COVID-19 (“post-COVID” hereafter) in the 14-month period from March 1, 2020, to April 30, 2021. Through ITSA, we were able to measure: (a) the baseline estimate of surgical volume at the start of the study period, (b) monthly trend during the pre-COVID period, (c) immediate change associated with the COVID-19 pandemic from March to April 2020, (d) monthly trend during the post-COVID period, and (e) differences in slope for pre- and post-COVID trends (Online Supplementary Table 2). To ensure that the ITSA models accounted for autocorrelation, we used the Cumby-Huizinga test to identify statistically significant lags, and adjustments were made accordingly.

Demographic and clinical characteristics were compared pre- and post-COVID using bivariate statistical analyses such as Wilcoxon rank-sum for medians and Pearson’s Chi-square test for categorical variables. Conclusions on statistical significance were made based on p-values with a preset level of significance *p* < 0.05. All statistical analyses were done on STATA 17 (StataCorp, College Station, TX, USA).

## Results

### Demographic & clinical characteristics

Across all participating sites, 6078 cases were recorded from January 2019 to April 2021, of which 2758 (45%) were pre-COVID and 3320 (55%) were post-COVID (Table 1). The median age of children who underwent surgery was 3.3 years pre-COVID and decreased to 2.7 years post-COVID (*p* < 0.001). Specifically, more neonates (9.4% vs 14.5%, *p* < 0.001) and fewer adolescents (12.7% vs 9.5%, *p* < 0.001) underwent surgery during the post-COVID period. In terms of socioeconomic data, 9.2% (561/6078) of the patients’ families reported their annual income and the median differed pre- and post-COVID ($750 vs. $1350 in United States dollars, *p* = 0.072), though this did not reach statistical significance.

**Table 1:** Demographic characteristics pre- vs post-COVID

	Pre *N* = 2758	Post *N* = 3320	Total *N* = 6078	*p*-value
Sex				0.39
Female	925 (33.8%)	1079 (32.7%)	2004 (33.2%)	
Male	1815 (66.2%)	2220 (67.3%)	4035 (66.8%)	
Age (year)	3.3 (0.8–8.6)	2.7 (0.4–7.4)	3.0 (0.6–7.9)	< 0.001*
Age group				
Neonate	254 (9.4%)	326 (14.5%)	580 (11.7%)	< 0.001*
Infant	824 (30.4%)	650 (28.9%)	1474 (29.7%)	0.223
Young child	709 (26.2%)	614 (27.3%)	1323 (26.7%)	0.4
School age	575 (21.2%)	448 (19.9%)	1023 (20.6%)	0.24
Adolescent	345 (12.7%)	215 (9.5%)	560 (11.3%)	< 0.001*
Sites				
Burkina Faso	1282 (46.5%)	1822 (54.9%)	3104 (51.1%)	< 0.001*
Ecuador	296 (10.7%)	220 (6.6%)	516 (8.5%)	< 0.001*
Nigeria	354 (12.8%)	430 (13.0%)	784 (12.9%)	0.893
Zambia	826 (29.9%)	848 (25.5%)	1674 (27.5%)	< 0.001*
Annual income ($)	750 (75–5592)	1350 (270–5592)	750 (75–5592)	0.072

Data are presented as *n* (%) for categorical measures and median (interquartile range) for continuous measures

*Statistically significant at alpha of *p* < 0.05

Regarding clinical characteristics (Table 2), a statistically significant decrease in elective surgery from 55 to 52% and increase in emergency surgery from 45 to 48% occurred from pre- to post-COVID periods (*p* = 0.016). Additionally, postoperative outcomes also improved from pre- to post-COVID, with lower rates of sepsis (1.5% vs 0.3%, *p* < 0.001), surgical site infections (5.8% vs 1.3%, *p* < 0.001), and mortality (3.1% vs 1.6%, *p* < 0.001). The average length of stay decreased from 4.1 to 3.5 days (*p* = 0.009), and the rate of reoperations dropped from 19.7 to 16.0% (*p* < 0.001). Safety checklist usage also decreased from 44.9 to 41.0% (*p* = 0.003). Patients classified as American Society of Anesthesiologists (ASA) status I increased from pre- to post-COVID (65.6% vs. 69.3%, *p* = 0.003) while those that were ASA III decreased (7.8% vs. 4.5%, *p* < 0.001). In terms of diagnoses, there were statistically significant increases in those categorized as general surgery (32% vs 34%, *p* = 0.048) and “other” (10% vs 17%, *p* < 0.001), and decreases in infection (19% vs 16%, *p* < 0.001) and trauma (16% vs 12%, *p* < 0.001).

**Table 2:** Demographic characteristics pre- vs post-COVID

	Pre *N* = 2758	Post *N* = 3320	Total *N* = 6078	*p*-value
Type of surgery				0.016*
Emergency	1238 (45.2%)	1599 (48.3%)	2837 (46.9%)	
Elective	1501 (54.8%)	1711 (51.7%)	3212 (53.1%)	
Disease category				
Congenital	498 (18.1%)	543 (16.4%)	1041 (17.1%)	0.081
General	869 (31.5%)	1125 (33.9%)	1994 (32.9%)	0.048*
Infection	514 (18.7%)	515 (15.5%)	1029 (17.0%)	0.001*
Oncology	147 (5.3%)	177 (5.3%)	324 (5.3%)	0.995
Trauma	447 (16.2%)	390 (11.8%)	837 (13.8%)	< 0.001*
Other	280 (10.2%)	565 (17.0%)	845 (13.9%)	< 0.001*
ASA class				
I	1723 (65.6%)	2173 (69.3%)	3896 (67.6%)	0.003*
II	671 (25.6%)	796 (25.4%)	1467 (25.5%)	0.882
III	206 (7.8%)	140 (4.5%)	346 (6.0%)	< 0.001*
IV	25 (1.0%)	26 (0.8%)	51 (0.9%)	0.62
Safety checklist	1232 (44.9%)	1354 (41.0%)	2586 (42.8%)	0.003*
Pre-op sepsis	312 (11.4%)	206 (6.2%)	518 (8.6%)	< 0.001*
Post-op sepsis	42 (1.5%)	10 (0.3%)	52 (0.9%)	< 0.001*
Surgical site infection	160 (5.8%)	43 (1.3%)	203 (3.4%)	< 0.001*
Reoperation	539 (19.7%)	526 (16.0%)	1065 (17.7%)	< 0.001*
LOS (days)	4.1 (3.7–4.5)	3.5 (3.2–3.7)	3.7 (3.5–4.0)	0.009*
Mortality	75 (3.1%)	50 (1.6%)	125 (2.3%)	< 0.001*

Data are presented as *n* (%) for categorical measures and median (interquartile range) for continuous measures

*Statistically significant at alpha of *p* < 0.05

### Surgical volume

**Fig. 1:**
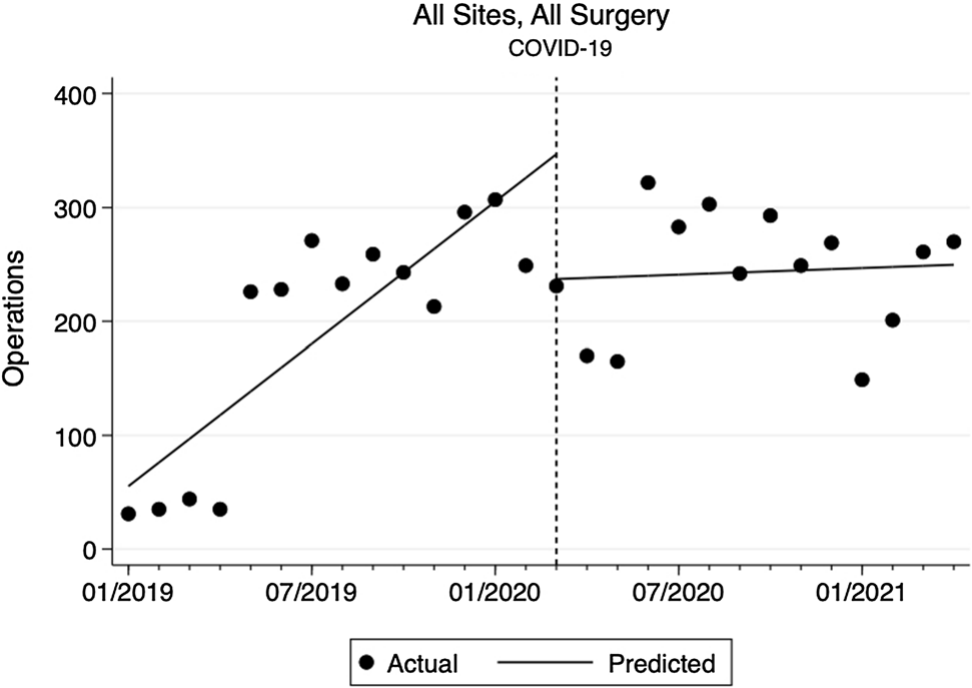
Changes in monthly total surgical volume at all sites pre- and post-COVID

**Fig. 2:**
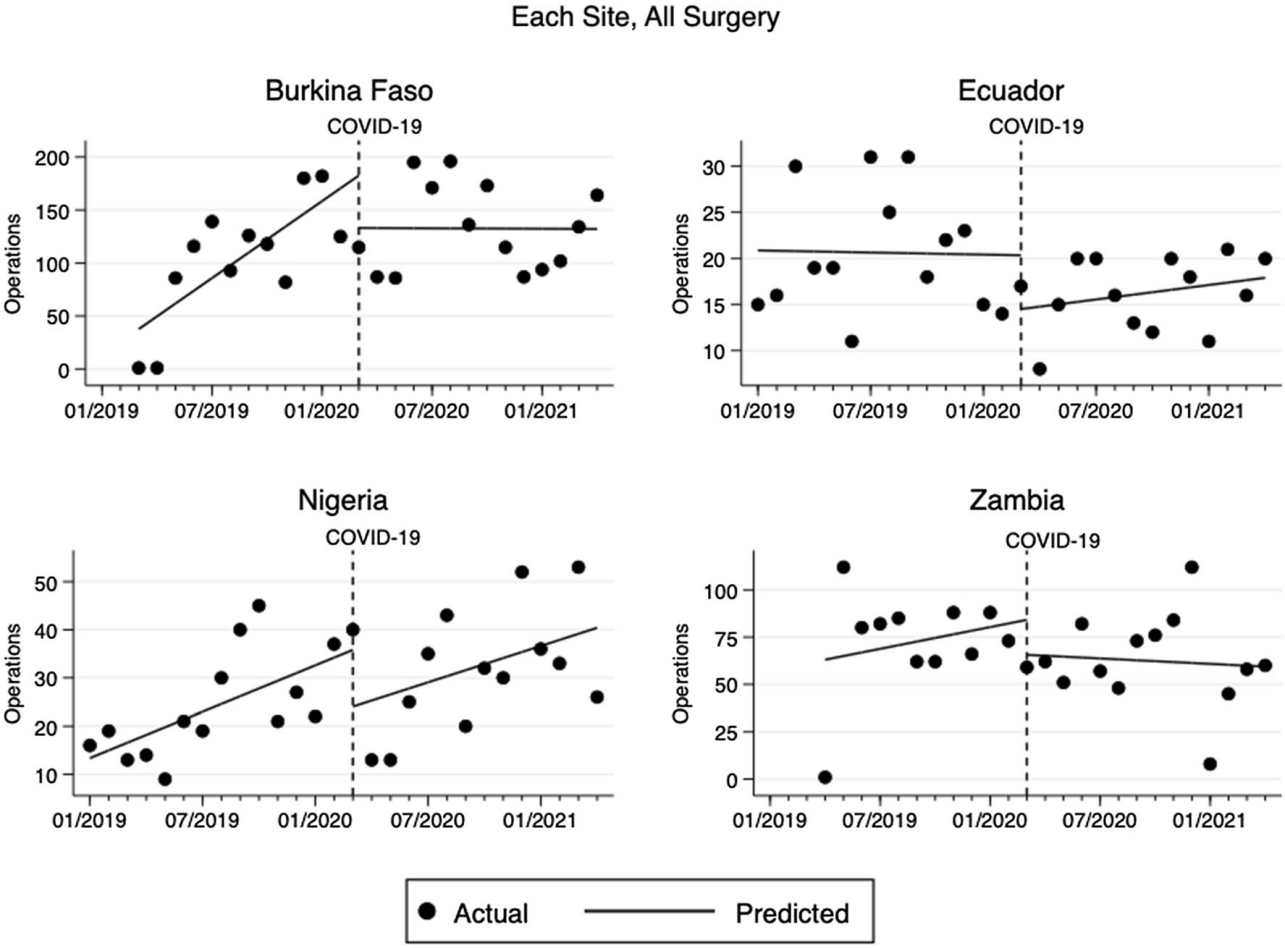
Changes in monthly total surgical volume at each site pre- and post-COVID

Burkina Faso accounted for nearly half of the total surgical volume (51%), followed by Zambia (30%), Nigeria (13%), and Ecuador (9%). From January 2019 to February 2020, the surgical volume across the sites altogether increased by 21 cases/month (95% CI 13.5 to 28.2, *p* < 0.001) from a baseline of 55 cases/month (Fig. 1). With the COVID-19 pandemic, the surgical volume dropped by 110 cases (95% CI − 196 to − 24, *p* = 0.014), which was a 32% decrease, from March 2020 to April 2020. In the subsequent post-COVID period, the surgical volume recovered at a rate of 1 additional case/month (95% CI − 6.3 to 8.2, *p* = 0.784). This reduction in the rate of change from pre-COVID trend by 20 cases/month (95% CI − 30 to − 10, *p* < 0.001) was statistically significant. Similar patterns in total surgical volume emerged individually at each site (Fig. 2). Of note, the surgical volume returned to pre-COVID level in Nigeria only.

**Fig. 3:**
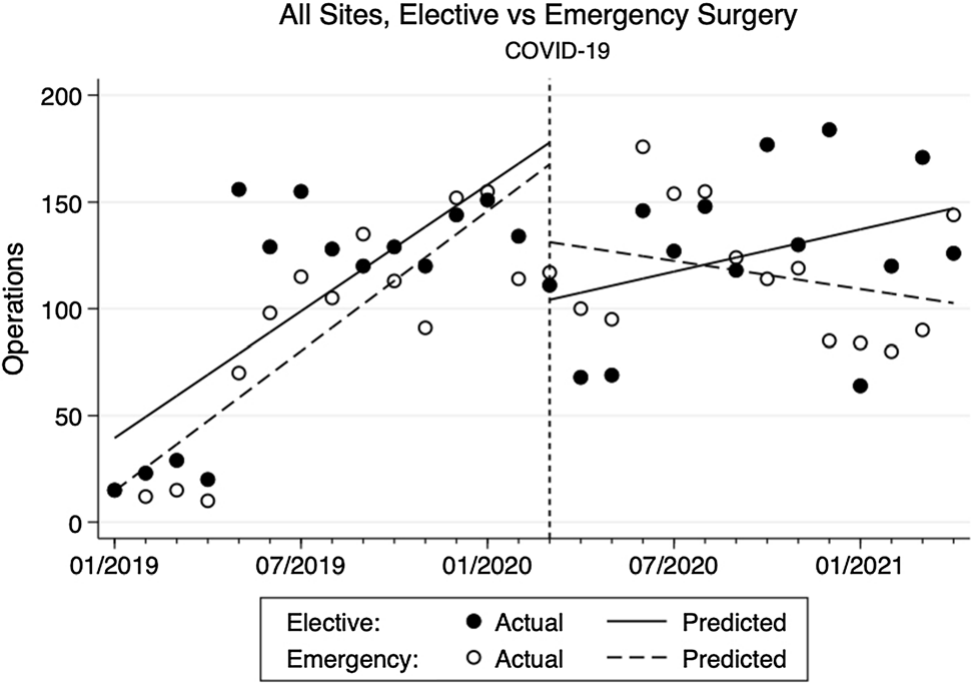
Changes in monthly elective and emergency surgical volume at all sites pre- and post-COVID

**Fig. 4:**
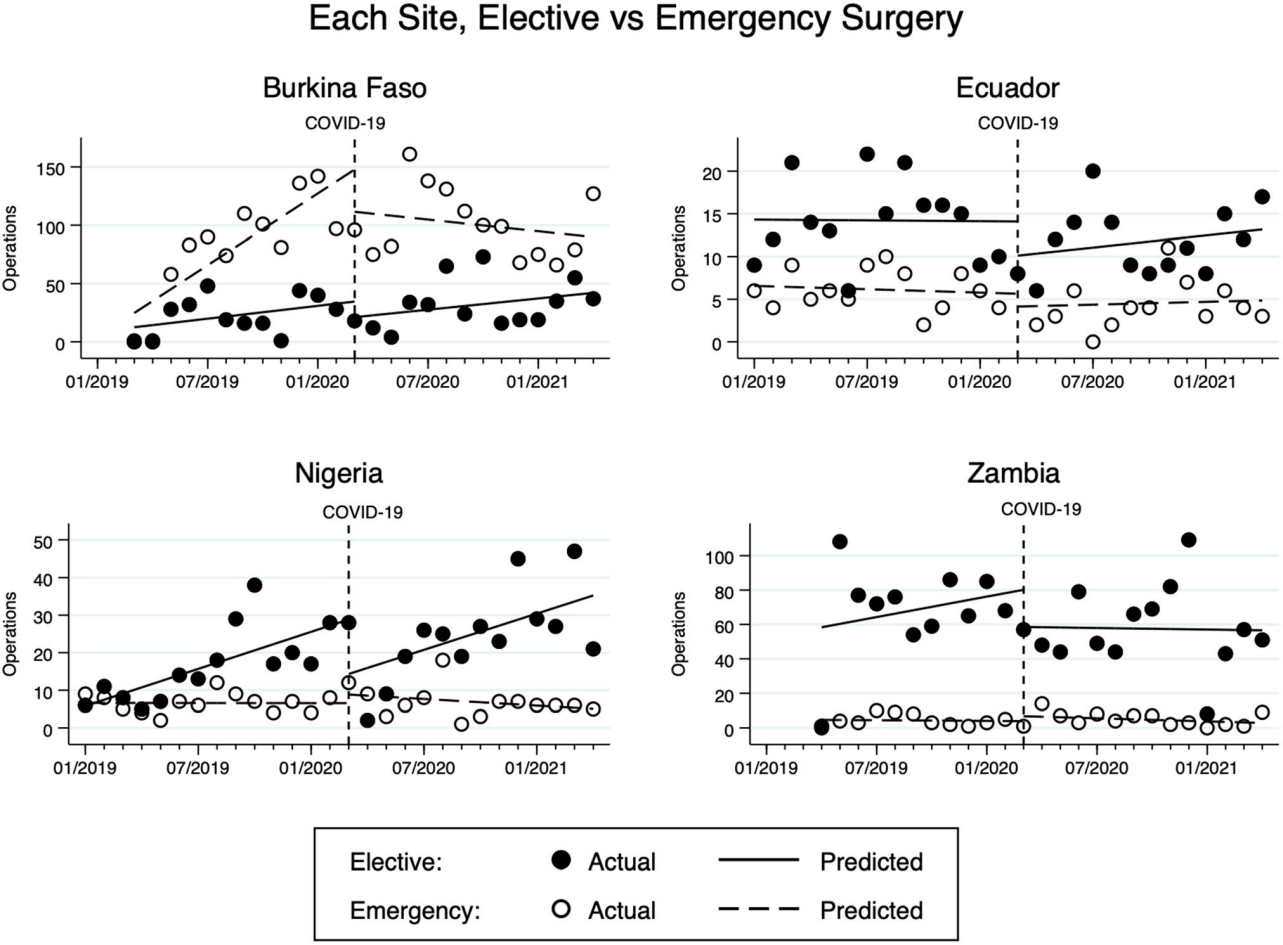
Changes in monthly elective and emergency surgical volume at each site pre- and post-COVID

The change in the type of surgical cases also differed (Fig. 3). Both elective and emergency surgical volume increased by 10 cases/month (95% CI 6 to 14, *p* < 0.001) and 11 cases/month (95% CI 8 to 14, *p* < 0.001), respectively, during pre-COVID. In the immediate month following COVID-19 from March to April 2020, the volume dropped by 73 cases (95% CI − 117 to − 30, *p* = 0.002) for elective surgery and 36 cases (95% CI − 71 to − 1, *p* = 0.043) for emergency surgery. During the post-COVID period, the elective surgical volume recovered at a rate of 3 cases/month (95% CI − 2 to 8, *p* = 0.175), while emergency surgical volume decreased by 2 cases/month (95% CI − 6 to 2, *p* = 0.22). Although these post-COVID trends were not statistically significant, the reductions in the rate of change from the pre-COVID trend by 7 cases/month (95% CI − 13 to − 1, *p* = 0.038) for elective surgery and by 13 cases/month (95% CI − 30 to − 10, *p* < 0.001) for emergency surgery were statistically significant. On further sub-analysis at each site (Fig. 4), similar trends and changes were again found for elective and emergency surgeries, but lacked statistical significance.

## Discussion

This study investigated the effects of the COVID-19 pandemic on the pediatric surgical volume in four hospitals in Burkina Faso, Nigeria, Ecuador, and Zambia. There were several statistically significant changes in the demographic and clinical characteristics of the patients from pre- to post-COVID. For instance, the median age of patients decreased by 0.6 years, and further sub-analysis by age groups revealed more neonates and fewer adolescents presenting during the pandemic. This was likely due to neonates already being present in the hospital following their births and neonatal care being prioritized in tertiary referral hospitals in LMIC as other available healthcare facilities may not have the capacity to provide neonatal surgical care. For adolescents, the decrease in the presentation may reflect a reduction in road traffic injuries, the major contributor of surgical morbidity and mortality in this age group in LMIC, due to lockdown policies seen worldwide [25–27]. Additionally, median family annual income was $600 higher during the pandemic, which suggests that poorer patients were less likely to seek care during the pandemic. This difference approached statistical significance and may have been underpowered as only a small proportion of patient families volunteered socioeconomic information.

Clinically, patients who presented for surgery during the pandemic were healthier with lower ASA class and had better outcomes with > 50% reductions in post-operative sepsis, surgical site infection, mortality, and smaller reductions in the rate of reoperation and length of stay. These improvements likely reflect a change in the patient population where sicker patients may not be getting the needed care due to increased barriers to access healthcare during the pandemic [28, 29]. Our findings add to the growing evidence for hidden morbidity and excess mortality during the pandemic from patients delaying care and potentially dying at home, especially in rural and underserved areas [30, 31].

As for surgical volume, there were sharp declines in the immediate month following the pandemic's start from March to April 2020. This finding contrasts with the steady rise in the total number of operations at all sites during the pre-COVID period, which could be due to KidsOR and its efforts to increase surgical capacities at these sites by providing dedicated surgical rooms or specialized equipment. By case type, elective surgeries fell sharply in the months following the pandemic, consistent with cancellations and postponements seen worldwide. Importantly, these findings address the limitations in international studies that found the highest rates of elective surgery cancellations in HIC likely due to limited data and participation from LMIC [8]. On the other hand, although emergency surgical volume similarly decreased, it has continued to decrease during the post-COVID period. This finding may be consistent with those in other studies where hospitals worldwide that experienced decreases in emergent surgeries during the pandemic [32–37]. Again, we believe this may be due to sicker patients avoiding medical care or facing insurmountable challenges in accessing care during the pandemic, but more research is needed.

Additionally, the overall surgical volume has been slow to recover in the subsequent months during the pandemic. For all sites, the total number of surgeries has not rebounded back to pre-COVID volume more than a year after the start of the pandemic. Although this study did not directly measure delayed or canceled operations, this finding likely exacerbates the pre-existing pediatric surgical backlogs in LMIC [38–40]. Furthermore, the slow recovery rate in surgical volume may be generalizable to other locations in Sub-Saharan Africa and other LMIC. Greater human and physical resources in HIC allow for healthcare systems in HIC to manage COVID-19 surges. Meanwhile, healthcare systems have been overwhelmed in LMIC and worsened by inequities in resources such as personal protective equipment and vaccines [41–45]. The findings of our study are particularly concerning as many LMIC have reported surges of COVID cases in recent months [46]. Lastly, the lack of easily accessible data in LMIC has made it difficult to assess the true impact of COVID-19 and track trends. These factors all combine to not only prolong the reduction in surgical capacity in LMIC but also make it difficult to coordinate and plan recovery strategies.

Given these findings, innovative strategies are needed to mitigate the impact. On a community level, outreach programs to increase access to healthcare providers or transportation policies designed for patients with acute medical needs could help address the hidden morbidity. Although widely used in HIC during the pandemic, telehealth has been underutilized in LMIC and should be further utilized to increase healthcare access [47–49].

On a broader level, strategies such as task sharing that had been adopted in LMIC to help bridge the gaps in surgical care could be expanded [50, 51]. For example, in pediatric surgery, task sharing may be a feasible alternative to help meet surgical demand for certain simple procedures such as hernia repairs, which medical officers can perform. The impact on the surgical and anesthesia workforce also needs to be considered, especially given that a substantial amount of the surgical workforce has been infected or died in LMIC [52, 53]. Provider shortages will likely continue due to high-risk work environments in these areas [54]. In the short-term, solutions such as fast-tracking medical and nursing training as well as virtual didactics have been suggested [55, 56].

Global partnerships can also play a substantial role in recovery from the pandemic. For instance, KidsOR has dedicated support for infrastructure, workforce strengthening, and local data platforms in partnership with multiple stakeholders, including hospitals, universities, and regional professional organizations. Similarly, other organizations such as Smile Train and Lifebox have continued to provide funding, resources, and training to ensure the continued provision of safe surgical care in LMIC [57, 58]. As the pandemic continues, long-term and systemic strategies on a global scale will need to be employed.

Our study has several limitations. First, the retrospective nature of the analyses did not allow us to control for potential confounding factors, and therefore, associations that other local and regional events may have influenced. For example, the initial lockdown period was used as the sole cut-off between pre- and post-COVID periods without factoring in subsequent partial lockdowns and fluctuations in pandemic intensity. Second, the participating study sites have differences such as the type of hospital (e.g., public vs. private), patient population (e.g., urban vs. rural), and overall surgical volume (e.g., high in Burkina Faso vs. low in Ecuador). Given these differences, direct comparisons by type of surgery and between the sites could not be made. Third, the ITSA model lacked statistical significance at the individual site level and by case type due to fewer cases on stratification. Fourth, there was significant variation in data availability on socioeconomic variables due to the sensitive nature of these questions, such that only 9% of the participants responded with their annual incomes. Lastly, the inclusion criteria for the sites required consistent data collection from 2019 to 2021, and as such, four of the partner hospitals with missing data were excluded. These sites may have been affected by the pandemic more and may not have had the capacity to enter data in a timely fashion.

## Conclusion

The decreases in surgical volume without evidence of sustained recovery across multiple LMIC hospitals demonstrates the persistent effects of the pandemic and provides evidence that the collateral damage of the pandemic on health services has extended to children’s surgery. Healthier and wealthier patients undergoing surgery with improved outcomes post-pandemic also raise concerns about hidden morbidity and mortality at the population level, especially among those with less resources. Overall, these findings call for a renewed commitment to equity, global partnerships, and innovative resource mobilization to mitigate the pandemic's impact on surgical services for children now and for future public health crises.

## Supplementary Information

Below is the link to the electronic supplementary material.

Supplementary file1 (PDF 284 kb)
