# Supplementary material for: Impact of the COVID-19 Pandemic on Pediatric Surgical Volume in Four Low- and Middle-Income Country Hospitals: Insights from an Interrupted Time Series Analysis
Source: World J Surg. 2022 Mar 10;46(5):984–93. doi: 10.1007/s00268-022-06503-2 (PMC8908743; doi:10.1007/s00268-022-06503-2)
Supplement: Supplementary file 1 — Supplementary file1 (PDF 284 kb) [file 268_2022_6503_MOESM1_ESM.pdf]

Electronic Supplementary Material

Title: Impact of the COVID-19 Pandemic on Pediatric Surgical Volume in four Low- and Middle-Income Country Hospitals: Insights from an Interrupted Time Series Analysis

Journal: World Journal of Surgery

Authors:
Paul Park, MS[1,2], Ruth Laverde, BS[1,2], Greg Klazura, MD[2,3], Ava Yap, MD, MHS[2,4], Bruce Bvulani, MBChB[5], Bertille Ki, MD[6], Toussaint W Tapsoba, MD[6], Emmanuel A Ameh, MBBS[7], Maryrose Osazuwa, MBBS[8], Michele Ugazzi, MD[9], José Daza, MD[9], Emma Bryce, MPH[10], David Cunningham[10], Doruk Ozgediz, MD, MSc[2,4]

[1] *University of California, San Francisco, School of Medicine, San Francisco, CA, USA*
[2] *University of California, San Francisco, Center for Health Equity in Surgery and Anesthesia, San Francisco, CA, USA*
[3] *Loyola University Health System, Department of Surgery, Maywood, IL, USA*
[4] *University of California, San Francisco, Department of Surgery, San Francisco, CA, USA*
[5] *University Teaching Hospital of Lusaka, Department of Paediatric Surgery, Lusaka, Zambia*
[6] Centre Hospitalier Universitaire Pédiatrique Charles De Gaulle*, Department of Paediatric Surgery, Ouagadougou, Burkina Faso*
[7] *National Hospital, Abuja, Division of Paediatric Surgery, Abuja, Nigeria*
[8] *National Hospital, Abuja, Department of Anesthesia, Abuja, Nigeria*
[9] *Hospital de los Valles, Division of Pediatric Surgery, Quito, Ecuador*
[10] *Kids Operating Room, Edinburgh, Scotland, United Kingdom*

Correspondence to: Paul Park
University of California, San Francisco, School of Medicine
513 Parnassus Ave, Suite S-224, San Francisco, CA 94143, USA
paul.park@ucsf.edu; +1 (858) 880-5151

Supplemental Table 1 Variables in the Data Collection Protocol

Variable	Category	Variable Label
record_id	demographics	Record ID
site_country	demographics	Country
study_id	demographics	Study ID
dob	demographics	Date of birth (if known)
age_number	demographics	Age
age_units	demographics	Age Unit
sex	demographics	Sex
weight	demographics	Weight (kilograms)
admit_date	demographics	Date of admission
surgery_date	demographics	Date of surgery
transfer	demographics	Was the patient transferred from another health center?
existing_patient	demographics	Is this a new entry for an existing patient who has previously had an operation in this OR?
staged	demographics	Is this operation part of a planned or staged procedure?
time	demographics	Time of operation
prematurity	demographics	Was the patient born prematurely (born before 37 weeks of gestation)?
elective	surgeon	Was this an elective (planned) operation?
diagnosis_1	surgeon	Diagnosis - 1
diagnosis_2	surgeon	Diagnosis - 2
diagnosis_3	surgeon	Diagnosis - 3
diagnosis_4	surgeon	Diagnosis - 4
other_anorectal	surgeon	Specify Other Anorectal Disease
other_intestinal_atresia	surgeon	Specify Other Intestinal Atresia
other_esophageal_atresia	surgeon	Specify Other Esophageal Atresia
hypospadias_subtype	surgeon	Specify Hypospadias Subtype
other_urogenital	surgeon	Specify Urogenital Malformation
other_heart	surgeon	Specify Congenital Heart Disease
other_msk	surgeon	Specify Musculoskeletal Malformation
other_mmc	surgeon	Specify Spina Bifida Type
other_teratoma	surgeon	Specify Teratoma Site
other_abd_mass	surgeon	Specify Abdominal Mass or Tumor
other_ovarian	surgeon	Specify Ovarian Mass or Tumor
other_testicular	surgeon	Specify Testicular or Scrotal Mass or Tumor
other_soft_tissue	surgeon	Specify Soft Tissue Mass or Tumor
other_extremity	surgeon	Specify Extremity Mass or Tumor
other_lymphoma	surgeon	Specify Myeloproliferative Disorder
other_trauma	surgeon	Specify Trauma
other_burn	surgeon	Specify Burn Site
other_abscess_type	surgeon	Specify Skin & Soft Tissue Infection Type
other_abscess_location	surgeon	Specify Skin & Soft Tissue Infection Location
other_necfasc_type	surgeon	Specify Necrotizing Soft Tissue Infection Type
other_necfasc_location	surgeon	Specify Necrotizing Soft Tissue Infection Location
other_intestinal_perf	surgeon	Specify Other Intestinal Perforation
other_neonatal_sepsis	surgeon	Specify Neonatal Sepsis
post_op_complications	surgeon	Specify Post Operative Complication
other_post_op	surgeon	Specify Other Post Operative Complication

other_unspecified	surgeon	Specify Other
other_foreign_body	surgeon	Specify Other Foreign Body Location
other_cns	surgeon	Specify CNS Mass or Tumor
other_thoracic	surgeon	Specify Thoracic Mass or Tumor
other_airway	surgeon	Specify Airway Disorder
other_infectious	surgeon	Specify Infectious Disease
other_infectious_specify	surgeon	Specify Other Infectious Disease
other_ldn	surgeon	Specify Lymphadenopathy
procedure_1	surgeon	Procedure - 1
procedure_2	surgeon	Procedure - 2
procedure_3	surgeon	Procedure - 3
procedure_4	surgeon	Procedure - 4
procedure_5	surgeon	Procedure - 5
specify_small_bowel_re sect	surgeon	Specify Small Bowel Segment
other_small_bowel_seg	surgeon	Specify Other Small Bowel Segment
pull_through_type	surgeon	Specify Pull-Through Type
specify_anoplasty	surgeon	Specify Other Anoplasty (ex: cloacal exstrophy repair)
specify_gastroschisis	surgeon	Specify Gastroschisis Repair Type
specify_omphalocele	surgeon	Specify Omphalocele Repair Type
specify_choledochal	surgeon	Specify Choledochal Cyst Excision Type
specify_hernia	surgeon	Specify Herniorrhaphy Site
specify_hypospadias	surgeon	Specify Hypospadias Reconstruction Technique
specify_urogenital	surgeon	Specify Complex Urogenital Repair Procedure
specify_abdominal	surgeon	Specify Abdominal Mass Site
specify_skin_graft	surgeon	Specify Skin Graft Site
specify_osteo	surgeon	Specify Osteomyelitis Site
specify_thoracic	surgeon	Specify Thoracic Procedure
specify_gastric	surgeon	Specify Gastric Procedure
specify_hepatic	surgeon	Specify Hepatic Procedure
specify_pancreatic	surgeon	Specify Pancreatic Procedure
specify_splenic	surgeon	Specify Splenic Procedure
specify_kidney	surgeon	Specify Kidney Procedure
specify_adrenal	surgeon	Specify Other Adrenal Procedure
specify_vascular	surgeon	Specify Vascular Procedure
specify_heart	surgeon	Specify Congenital Heart Repair
specify_msk	surgeon	Specify Musculoskeletal Procedure
specify_cns	surgeon	Specify Central Nervous System Procedure
specify_other	surgeon	Specify Other Procedure
primary_surgeon	surgeon	Primary surgeon type
assistant_surgeons	surgeon	Assistant Surgeons (select all that apply)
surgical_trainee_present	surgeon	Was a surgical trainee present?
essential_equipment_s	surgeon	Were necessary surgical equipment missing?
missing_list_s	surgeon	Which resources were not available? (select all that apply)
other_equipment_s	surgeon	Specify other equipment not available
other_resources_s	surgeon	Specify other resources not available
laparoscopy_available	surgeon	Was laparoscopy available for this operation?
laparoscopy_used	surgeon	Was laparoscopy used for this operation?

lap_would_have_used	surgeon	Would you have considered using laparoscopy if it was available?
laparoscopy_issues	surgeon	Were there any problems using laparoscopy during this case?
lap_issues_description	surgeon	Describe the problems with laparoscopy that were encountered:
lap_why_not	surgeon	Why wasn't laparoscopy used during this operation?
lap_why_not_desc	surgeon	List other reason laparoscopy wasn't used:
anaesthesia_provider	anaesthetist	Anaesthesia provider (select all that apply)
anaesthesia_type	anaesthetist	Anaesthesia type (select all that apply)
anaesthesia_gen_specify	anaesthetist	Specify the type of general anesthesia used? (Select all that apply)
anaesthesia_trainee	anaesthetist	Was an anaesthesia trainee present?
essential_equipment_a	anaesthetist	Were necessary anaesthesia equipment and / or drugs missing?
missing_list_a	anaesthetist	Which resources were not available? (select all that apply)
other_resources_a	anaesthetist	Specify other resources not available
asa	anaesthetist	ASA Class
preop_temp	anaesthetist	Pre-Operative Temperature (degrees Celsius)
sepsis	anaesthetist	Sepsis in 48 hours prior to surgery?
sepsis_specify	anaesthetist	How was sepsis diagnosed? (select all that apply)
sirs_criteria_1	anaesthetist	SIRS criteria by age group
blood_transfusion	anaesthetist	Did the patient receive a blood transfusion?
blood_transfusion_specify	anaesthetist	Specify when the patient received blood transfusion. (select all that apply)
oxygen	anaesthetist	Did the patient receive supplemental oxygen?
oxygen_specify	anaesthetist	Specify when the patient received oxygen. (select all that apply)
pre_op_abx	anaesthetist	Did the patient receive antibiotics?
pre_op_abx_specify	anaesthetist	Specify when the patient received antibiotics. (select all that apply)
safety_checklist	anaesthetist	Safety Checklist Used in OR?
postop_temp	anaesthetist	Post-Operative Temperature (degrees Celsius)
discharge_date	discharge	Date of discharge
icu	discharge	Did the patient require ICU care post-operatively?
icu_no	discharge	Did the patient require higher level of care, but ICU was not available?
icu_duration	discharge	How many days was the patient admitted to the ICU?
post_op_abx	discharge	Antibiotics given post-operatively?
post_op_sepsis	discharge	Post-operative sepsis?
post_op_sepsis_specify	discharge	How was sepsis diagnosed? (select all that apply)
sirs_criteria_2	discharge	SIRS criteria by age group
wound_infection	discharge	Post operative surgical site infection?
re_operation	discharge	Did the patient need another operation this admission?
discharge_status	discharge	Status at discharge
death_on_table	discharge	Was this an on-table death?
wait_time	socioeconomics	How long did the child wait to have the operation?
hospital_cost	socioeconomics	Out of pocket DIRECT medical cost of hospitalization (hospital stay, procedures, medical and diagnostic tests, medications, supplies, etc.)
travel_cost	socioeconomics	Out of pocket INDIRECT medical cost of hospitalization (transportation, feeding, employing an attendant, etc.)
borrow_money	socioeconomics	Did your household have to borrow money to pay for this hospitalization?
sell_possessions	socioeconomics	Did your household have to sell any land or possessions to pay for this hospitalization?
lost_wages	socioeconomics	Did your household lose wages as result of this hospitalization?
lost_job	socioeconomics	Did you or anyone in your household permanently lose a job as a result of this hospitalization?

annual_income	socioeconomics	Annual family income BEFORE this hospitalization
subsidy	socioeconomics	Did your child receive any government subsidy to help pay for this operation?
subsidy_value	socioeconomics	What was the approximate monetary value of this subsidy?
travel_time	socioeconomics	How long did it take you to reach the hospital when you started travelling?

Supplemental Table 2 Interrupted Time Series Analysis Output

	**Total**			**Elective**			**Emergency**		
	Volume (cases/ month)	***95% CI***	***p-value***	***Volume (cases/ month)***	***95% CI***	***p-value***	***Volume (cases/ month)***	***95% CI***	***p-value***
**All Sites**									
Baseline	55.3	-7.9, 118.5	0.083	40.7	-1.5, 82.8	0.058	15.8	-11.9, 43.5	0.251
Pre-COVID Δ	20.8	13.5, 28.2	**<0.001***	9.9	5.6, 14.2	**<0.001***	10.9	7.6, 14.3	**<0.001***
Immediate Δ	-109.8	-195.5, -24.2	**0.014***	-73.3	-116.6, -30.0	**0.002***	-36.0	-70.9, -1.2	**0.043***
Post-COVID Δ	1.0	-6.3, 8.2	0.784	3.2	-1.5, 7.9	0.175	-2.3	-6.2, 1.5	0.220
Pre- vs Post-	-19.9	-30.2, -9.6	**0.001***	-6.7	-13.1, -0.4	**0.038***	-13.3	-19.1, -7.4	**<0.001***
**Burkina Faso**									
Baseline	37.7	-7.1, 82.5	0.095	28.3	15.4, 41.2	**<0.001***	25.2	-5.6, 56.0	0.104
Pre-COVID Δ	12.1	6.2, 18.0	**<0.001***	-0.2	-2.5, 2.2	0.878	10.2	5.9, 14.6	**<0.001***
Immediate Δ	-49.5	-100.6, 1.6	0.057	-5.0	-30.9, 20.9	0.692	-36.1	-71.4, -0.8	**0.045***
Post-COVID Δ	-0.1	-5.5, 5.3	0.977	1.6	-0.2, 3.4	0.078	-1.7	-5.6, 2.3	0.386
Pre- vs Post-	-12.1	-20.9, -3.4	**0.009***	1.8	-1.0, 4.6	0.205	-11.9	-18.6, -5.3	**<0.001***
**Ecuador**									
Baseline	20.9	13.8, 27.9	**<0.001***	14.3	10.6, 18.1	**<0.001***	6.6	4.5, 8.7	**<0.001***
Pre-COVID Δ	-0.04	-0.87, 0.80	0.927	0.03	-0.63, 0.68	0.929	-0.02	-0.30, 0.26	0.872
Immediate Δ	-5.8	-14.0, 2.3	0.154	-4.3	-11.3, 2.6	0.211	-1.9	-6.3, 2.5	0.391
Post-COVID Δ	0.26	-0.28, 0.81	0.330	0.21	-0.30, 0.69	0.385	0.03	-0.37, 0.42	0.892
Pre- vs Post-	0.30	-0.70, 1.30	0.541	0.18	-0.73, 1.09	0.69	0.05	-0.43, 0.53	0.838
**Nigeria**									
Baseline	13.4	7.9, 18.9	**<0.001***	6.8	3.5, 10.1	**<0.001***	7.6	4.8, 10.3	**<0.001***
Pre-COVID Δ	1.6	0.7, 2.5	**0.002***	1.6	0.9, 2.3	**<0.001***	-0.04	-0.4, 0.3	0.777
Immediate Δ	-11.8	-29.3, 5.8	0.179	-14.1	-28.6, 0.4	0.057	2.8	-2.2, 7.7	0.258
Post-COVID Δ	1.3	-0.6, 3.1	0.178	1.6	-0.1, 3.2	0.070	-0.4	-0.7, -0.02	**0.040***
Pre- vs Post-	-0.3	-2.4, 1.8	0.739	-0.1	-1.9, 1.8	0.951	-0.3	-0.8, 0.2	0.18
**Zambia**									
Baseline	63.1	8.6, 117.5	**0.025***	58.4	6.5, 110.2	**0.029***	6.5	2.4, 10.6	**0.004***
Pre-COVID Δ	1.9	-5.8, 9.6	0.61	2.0	-5.4, 9.3	0.58	-0.4	-1.0, 0.3	0.233
Immediate Δ	-18.5	-52.9, 15.8	0.275	-21.4	-55.1, 12.4	0.202	4.3	-1.9, 10.6	0.163
Post-COVID Δ	-0.5	-3.1, 2.1	0.705	-0.2	-2.8, 2.4	0.887	-0.3	-1.0, 0.4	0.340
Pre- vs Post-	-2.4	-10.5, 5.7	0.546	-2.2	-9.9, 5.6	0.569	0.1	-0.9, 1.0	0.916
